# Randomized Control Trial for Reduction of Body Weight, Body Fat Patterning, and Cardiometabolic Risk Factors in Overweight Worksite Employees in Delhi, India

**DOI:** 10.1155/2017/7254174

**Published:** 2017-11-29

**Authors:** Usha Shrivastava, Mahrukh Fatma, Smriti Mohan, Padam Singh, Anoop Misra

**Affiliations:** ^1^National Diabetes, Obesity and Cholesterol Foundation, New Delhi, India; ^2^Diabetes Foundation (India), New Delhi, India; ^3^Centre for Public Health India, New Delhi, India; ^4^Fortis C-DOC Hospital for Diabetes, Metabolic Diseases and Endocrinology, New Delhi, India

## Abstract

**Background:**

We studied the impact of the multicomponent interventions on body weight and cardiometabolic risk factors in overweight individuals working in corporate worksites.

**Methods:**

Overweight (BMI ≥ 23 kg/m^2^) subjects were recruited from four randomised worksites [two active intervention (*n*, recruited, 180, completed 156) and two control (*n*, recruited 130, completed 111)]. Intensive intervention was given at intervention worksite.

**Results:**

High prevalence (%) of obesity (90.9, 80.2), abdominal obesity (93.5, 84.3), excess skinfold thickness (70.3, 75.9), and low high-density lipoprotein cholesterol (HDL-c) levels (56.8, 63.7) were seen in the intervention and the control group, respectively. At the end of intervention, the following significant changes were observed in the intervention group: decrease in weight, BMI, waist circumference, serum triglycerides, and increase in HDL-c. Weight loss of more than 5% was seen in 12% and 4% individuals in the intervention and control groups, respectively. Most importantly, the sum of all the skinfold measurements (mm) in the intervention group decreased significantly more than the control group (12.51 ± 10.38 versus 3.50 ± 8.18, resp.).

**Conclusion:**

This multicomponent worksite trial showed a reduction in weight, excess subcutaneous fat, and cardiometabolic risk factors after 6 months of active intervention in overweight Asian Indians.

**Trial Registration:**

This trial is registered with NCT03249610.

## 1. Introduction

Overweight/obesity, type 2 diabetes mellitus (T2DM), and cardiovascular diseases (CVDs) are rapidly increasing in Asian Indians, and these are accompanied by multiple other cardiovascular risk factors including hypertension and dyslipidemia [1, 2]. Moreover, such dysmetabolic state occurs early and rapidly deteriorates from the second decade of life [3]. These conditions are majorly contributed by imbalanced diet (high carbohydrates, high saturated fats, low fiber, etc.), physically inactive lifestyle, and stress [4]. Specifically, some segments of the population (women and people belonging to middle and low socioeconomic strata) are increasingly becoming vulnerable to obesity and clustering of cardiovascular risk factors (metabolic syndrome) in India [5]. Many other sections of the population, such as corporate and industrial workforce, have not been adequately investigated.

The workforce in industrial and corporate sectors is increasing in India. Many of such individuals do desk-based jobs and commute in motorized vehicles for long distances leaving little time for physical activity. Moreover, for most of the days, their food intake during office hours is based on availability of often unhealthy food articles in office cafeteria. However, their health status with particular reference to cardiovascular risk factors remains sparsely researched. In a study done on two industrial units in South India (*n*, 2262, males), 67% was overweight/obese, 70% had abdominal obesity, 27% had hypertension, 30% had high cholesterol, and 16% had T2DM [6]. These data raise concern and increase need for more research in the context of cardiovascular risk factors in workforce in industrial and corporate sectors in India. In particular, food habits, physical activity, obesity measures, and metabolic status of people working in these sectors need to be ascertained [6].

Worksite intervention remains an effective way of reducing cardiometabolic risk factors. Improving health in the workforce should not only benefit the employee but also may increase productivity, thus benefiting the employer as well. Most of the intervention studies included changes in weight and body mass index (BMI) as outcome measures, while others have focused on physical activity and diet. Almost half of the all the studies were undertaken in the USA and the rest in Europe, Iceland, Canada, Australia, New Zealand, and Japan but very few have been conducted in India. In a systematic review, a total of 47 studies (*n* = at least 76,941) were included, comprising of 24 randomized controlled trials (RCTs), seven cluster randomized controlled trials, 12 nonrandomized, three cohort studies, and one-time series. The authors summarized that the worksite nutrition and physical activity programmes achieved modest improvements in employee weight status at 6 to 12 months of follow-up. Analysis of RCTs showed that the interventions resulted in a decrease in weight of 2.80 pounds (95% CI −4.63 to −0.96; nine studies) and a reduction in BMI of −0.47 kg/m^2^ (95% CI −0.75 to −0.19; six studies) compared to the controls. Importantly, these findings are applicable to both genders and across wide variety of worksites. While most studies have combined informational and behavioural strategies for diet and physical activity, lesser number is focused on different areas of work environment [7].

Because of paucity of data, specifically interventions in a randomized manner in Asian Indians working in a corporate setup, we conducted 6-month intervention trial on employees in the age group of 25–55 years at different worksites at New Delhi, India. The primary objective of our study was to observe the effect of multicomponent lifestyle interventions on weight loss, and the secondary objective was to observe the changes in body fat patterning and reduction of cardiometabolic risk factors in at-risk, overweight individuals.

## 2. Material and Methods

### 2.1. Sample Size Calculation

This was a prospective comparative study having an intervention group and a control group. The objective of the study was to test whether the intervention has resulted into desired improvements in the parameters as compared to the control groups. Of various indicators, the BMI was the main indicator for the study.

The following assumptions have been made in deciding the sample size:

- Mean change in the value of the parameters (BMI) for the intervention group (Δ_1_) = 0.50.
- Mean change in the value of the parameters (BMI) for the control group (Δ_2_) = 0.10.
- The standard deviation of the change in the parameters for the intervention group (*σ*_1_) = 1.0.
- The standard deviation of the change in the parameters for the control group (*σ*_2_) = 0.90.

Confidence level = 95% and power = 90%. The ratio of sample size (intervention versus control) = 1.5. For detecting the difference of 0.4 between groups as significant, the sample size works out as 160 for the intervention group and 107 for the control group. The study was planned so as to achieve this sample size; however, the realized sample size is 156 for the intervention group and 111 for the control group.

Sample size calculation was done by the following:

(1) $$\begin{array}{c} n=\frac{\left({\sigma_{1}}^{2}+{\sigma_{1}}^{2}\right){\left(Z_{\alpha }+Z_{\beta }\right)}^{2}}{{\left(\Delta_{1}-\Delta_{2}\right)}^{2}}. \end{array}$$

The above formula is for the equal sample size. If the sample size for larger group is *c* times of smaller group, a factor (*f*) is applied to the smaller group which is given by *f* = (*c* + 1)/2*c*, in this case, the sample size of the smaller group is *n*_1_ = *f*^∗^*n* and that of the larger group is *n*_2_ = *c*^∗^*n*_1_, where *Z_α_* is the value of the standard normal variate corresponding to *α* level of significance, *Z_β_* is the standard normal deviate for desired power, Δ is the mean change in the parameter, and *σ* is the standard deviation of the change in the parameter.

The analysis included profiling of subjects by different sociodemographic parameters. For the intervention as well as the control group, the average values with SD have been computed for baseline as well as endline. Importantly, the improvements in parameters [endline (postintervention) versus baseline] have been estimated along with standard errors to test for their significance using the paired *t*-test. For comparing the significance of change in parameters in the intervention group over and above the control group, two sample *t*-tests have been used. *p* value < 0.05 is considered statistically significant. SPSS software (IBM SPSS Statistics 24.0) has been used for statistical analysis.

### 2.2. Site and Subject Recruitment

Worksites were eligible if they had not hosted a weight loss or any other wellness programme during the past 6 months, were easily accessible by public transportation, and had the basic infrastructure to hold on-site programmes and physical activity training sessions. We projected two sites as active intervention sites and other two as delayed intervention sites, labelled as control worksites. We then selected 4 worksites (both the public and private), from the different sites across Delhi and National Capital Region. The trial was initiated in 2014 after obtaining certification from Institutional Review Board. Initially, we contacted the human resource department of the worksites to explain the project and also whether their site would be intervention or control.

**Figure 1:**
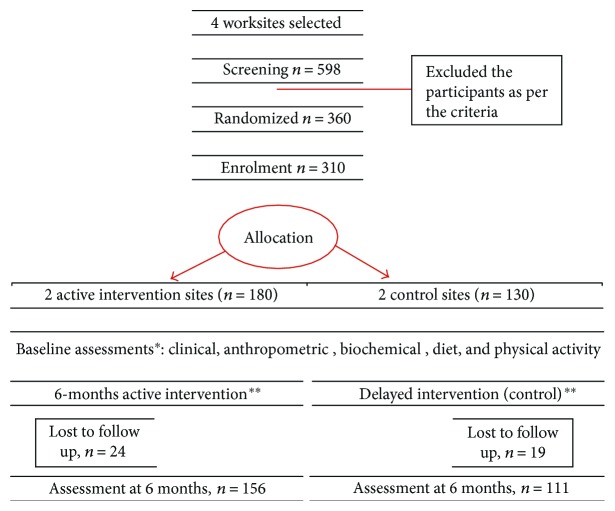
Screening and recruitment. ^∗^See text for assessment details. ^∗∗^See text for methodology of interventions.

Inclusion criteria were willingness to participate, age group of 25–55 yrs., and having BMI ≥ 23 kg/m^2^, that is, overweight subjects [8]. Exclusion criteria included previously diagnosed patients with diabetes, coronary artery disease, receiving medication(s) within the last one month which could potentially influence insulin secretion, insulin sensitivity, or weight, having severe end-organ damage or chronic diseases, and pregnant and lactating women, etc. After rapport building and sensitization, we screened 598 participants in both the groups then randomly selected 180 subjects in the intervention site and 130 in the control site by random-generated numbers (Figure 1). All subjects were fully informed about the purpose of the study, and a written informed consent (approved by Institutional Review Board) was obtained from each of them.

### 2.3. Interventions

A multicomponent intervention was implemented for 6 months for active intervention sites. The variables recorded at baseline and after intervention were demographic information, behavioural risk factors (tobacco and alcohol consumption), dietary intake, physical activity pattern, anthropometric measurements, blood pressure examination, and biochemical investigations. Tobacco consumption included data on self-reported duration, frequency, and quantity. Self-reported alcohol intake data were collected, and subjects were classified as present consumer, past consumer, and nonconsumer.

The sessions and trainings were led by an expert team of physicians, nutritionist, and physical trainer. The interventions were designed for participants only but the sessions were open to the whole worksite employees. Over a period of 6 months, different intervention strategies were followed to increase the employee awareness and improvement in knowledge, attitude, and practices to get the expected results. Participants in the intervention group received detailed sessions on the different topics related to healthy living, diet, and physical activity. Two sessions on each topic were conducted in the intervention sites every 15 days for the duration of 45–60 minutes. Subsequently, in these sessions, reinforcement and need-based advice were provided. The nutrition topics included healthy eating pattern and food articles, eating outside home, portion control, choice of oils, correct cooking methods, how to read the food labels, and eating during traditional Indian festive seasons based on dietary guidelines for Indians [9]. Two physical activity training sessions were given to explain the best practices in physical activity as per guidelines for Asian Indians [10] and to encourage them to continue physical activity supported by use of pedometers. Stress management sessions were also provided to the employees to cope up with the workplace and other kinds of stress. We tracked the compliance of lifestyle changes with the text messages from a smartphone, digital health platform, e-mails, and repeated phone calls. The participants in the control sites (delayed intervention) did not receive any kind of intervention but were given general health talk twice in six months.

### 2.4. Measurements

Blood pressure was recorded in a sitting position after 5 min rest with a mercury sphygmomanometer according to the standard guidelines. If one abnormal reading was observed, a second reading was recorded after 10 min of rest. For anthropometric measurements, weight was recorded to the nearest 0.1 kg and height to the nearest 0.1 cm. The BMI was calculated as weight (kg)/height (m)^2^. All the circumferences were measured by using flexible, nonstretchable tape. Waist circumference was measured midway between iliac crest and costal margin, and the hip circumference was measured at the maximum circumference of buttocks with the subject wearing minimum clothes. The mean of three readings of each was taken for the calculation of waist-hip ratio (W-HR). Biceps, triceps, subscapular, and suprailiac skinfolds were measured using Lange skinfold caliper (Beta Technology Inc., Santa Cruz, CA, USA). For biceps skinfold, with the right arm pendant, the fat pad was measured at the level of the nipple line, and triceps fat pad was measured midway between acromion process of scapula and olecranon process. Fat pads at the inferior angle of scapula, and superiorly on iliac crest directly in the midaxillary line, were measured for subscapular and suprailiac skinfolds. All skinfolds were measured to the nearest mm. A mean of three readings was recorded at each site.

Detailed dietary records were obtained from a subsample of subjects from the intervention group (*n*, 38) and the control group (*n*, 25). The food frequency questionnaire tool was used to measure habitual dietary intake [11]. The energy expenditure was assessed by using global physical activity questionnaire (GPAQ) [12] in a subsample of the intervention group (*n*, 85) and the control group (*n*, 80), and metabolic equivalent of task (MET) values were calculated.

### 2.5. Biochemical Investigations

Biochemical investigations included fasting blood glucose (FBG) and serum lipids [total cholesterol (TC), serum triglyceride (TG), and high-density lipoprotein cholesterol (HDL-c)] taken after 10–12 hours of overnight fasting and as previously analysed [13]. The value of low-density lipoprotein cholesterol (LDL-c) was calculated according to Friedewald's equation [14].

### 2.6. Definitions

Overweight and obesity were defined as BMI ≥ 23–24.9 kg/m^2^ and BMI ≥ 25 kg/m^2^, respectively [8]. Waist circumference cutoffs of ≥90 cm for males and ≥80 cm for females were considered an indicator of abdominal obesity [8]. High W-HR was defined as ≥0.90 in males and ≥0.80 in females [8]. Central (sum of subscapular and suprailiac) and peripheral (sum of biceps and triceps) skinfold thicknesses were calculated. The sum of all skinfolds (∑4SF) was also calculated, and a value of ≥53.8 mm was taken as excess skinfold since it indicates the presence of insulin resistance [15]. Impaired fasting glucose and T2DM were diagnosed according to the diagnostic criteria of the American Diabetic Association [16]. Metabolic syndrome was defined as the presence of three or more of the following abnormalities: abdominal obesity (defined as waist circumference ≥ 90 cm for men and ≥80 cm for women), raised systolic blood pressure (SBP) ≥ 130 mmHg or diastolic blood pressure (DBP) ≥ 85 mmHg, treatment of previously diagnosed hypertension, FBG ≥ 100 mg/dl treatment of, previously diagnosed T2DM, TG level ≥ 150 mg/dl or specific treatment for this lipid abnormality, HDL-c ≤ 40 mg/dl in males and ≤50 mg/dl in females, or specific treatment for this lipid abnormality [17].

## 3. Results

The baseline demographic characteristics of the participants in the intervention and the control group have been provided in Table 1. High prevalence of obesity, abdominal obesity, and high subcutaneous adiposity was seen in the control and intervention groups (Table 2). At the baseline, 95% individuals had at least one cardiovascular risk factor. There was no significant difference observed at the baseline in anthropometric, biochemical, and clinical parameters in both the groups (Table 3).

**Figure 2:**
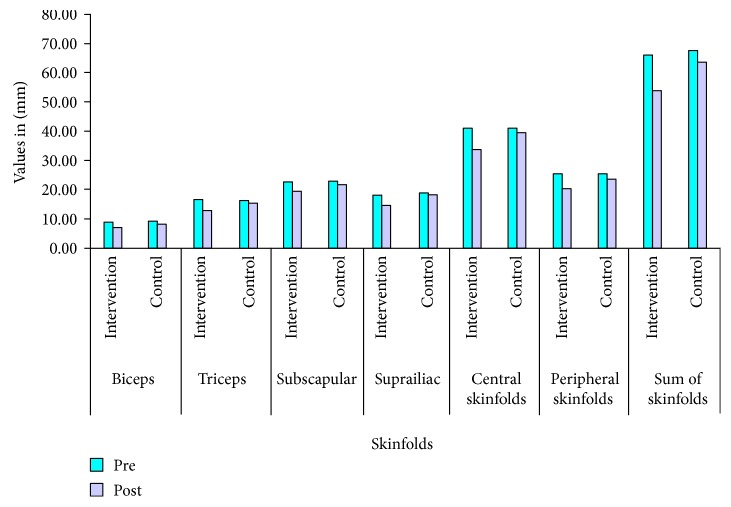
Changes in absolute values of skinfold thickness and sum of skinfolds after intervention. All the values are significant <0.05. Values placed above bars are absolute values of respective skinfolds in mm. See Table 5 for SD of skinfold measurements. ^∗^Central skinfold is the sum of subscapular and suprailiac skinfolds, peripheral skinfold is the sum of biceps and triceps skinfolds, and the sum of skinfolds is the sum of all the 4 skinfolds (∑4SF, biceps, triceps, subscapular, and suprailiac).

Intervention resulted with significant decrease in the mean values of weight, BMI, waist circumference, hip circumference, W-HR, all the four skinfolds (biceps, triceps, subscapular, and suprailiac), FBG, TG, and increase in HDL-c in the intervention group. There was no significant difference observed in the mean values of all these parameters except FBG and three skinfolds (biceps, triceps, and subscapular) in the control group after 6 months (Table 4). More than 5% weight loss was observed in 12% of the individuals in the intervention group as compared to only 4% of the individuals in the control group. Further, significant decrease in the skinfold measurements (mm) was seen in the intervention versus control group; biceps (1.8 ± 2.3 versus 0.95 ± 1.57), triceps (3.54 ± 3.67 versus 0.90 ± 2.70), subscapular (3.38 ± 4.21 versus 1.01 ± 3.35), and suprailiac (3.76 ± 3.79 versus 0.64 ± 3.53), respectively. Similarly, central and peripheral skinfolds (mm) in the intervention group decreased significantly as compared to the control group (7.14 ± 8.0 versus 1.65 ± 6.87 and 5.42 ± 5.92 versus 1.84 ± 5.92, resp.) (Table 4). As a result, the sum of all the skinfold measurements in the intervention group decreased significantly more than the control group (12.51 ± 10.38 versus 3.50 ± 8.18, resp.) (Figure 2). Further subscapular/triceps (SS/T) ratio increased from 1.37 to 1.48 in the intervention group, as opposed to the decrease in the control group (1.48 to 1.42).  Table 5 describes changes in the strata, from abnormal to normal, of obesity, abdominal obesity, excess skinfold thickness, lipids, and glucose values after intervention. Specifically, individuals with excess sum of all the skinfolds were reduced significantly in the intervention group as compared to the control group after intervention.

**Figure 3:**
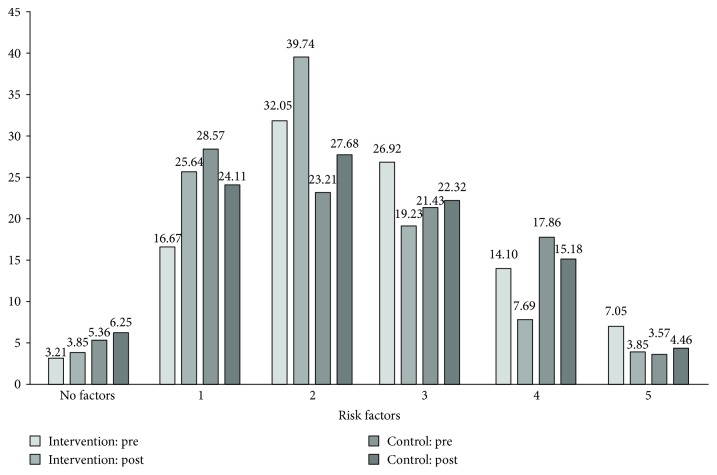
Changes in clustering of risk factors comprising metabolic syndrome before and after intervention. All the values are in the percentage. Please see text for definitions of risk factors.

Changes in the clustering of risk factors of metabolic syndrome have been shown in Figure 3. It is interesting to note that individuals with the reduction in the number of five, four, and three risk factors are more in the intervention group after intervention; status of many of those changed to two factors or one. It is of note that very few have reverted to nil or no risk factor profile in both the groups. Specifically, individuals with 3 risk factors decreased from 27% to 19% in the intervention group as compared to the increase from 21% to 22% in the control group. Individuals with four factors decreased more in the intervention as compared to the control groups (14% to 8% versus 18% to 15%, resp.). Similarly, in the intervention group, 5 risk factors decreased from 7% to 3% individuals but remained the same (from 4% to 5%) in the control group.

**Figure 4:**
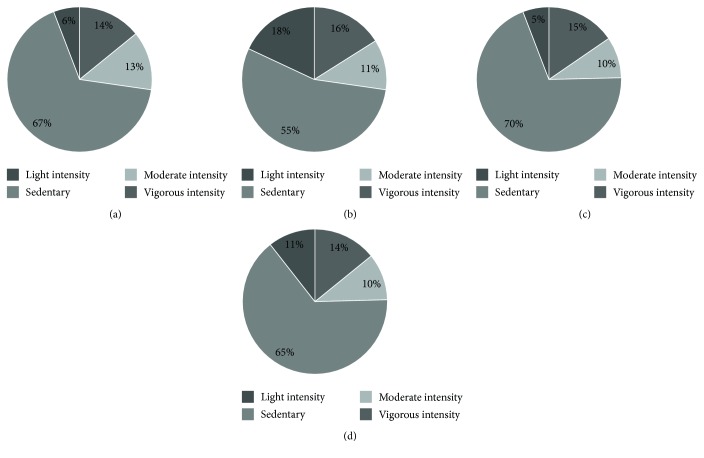
Change in physical activity profile after intervention. Showing physical activity profile. (a) Preintervention in intervention group (*n*, 85). (b) Postintervention in the intervention group (*n*, 80) (c) Preintervention in the control group (*n*, 85). (d) Postintervention in the control group (*n*, 80).

**Figure 5:**
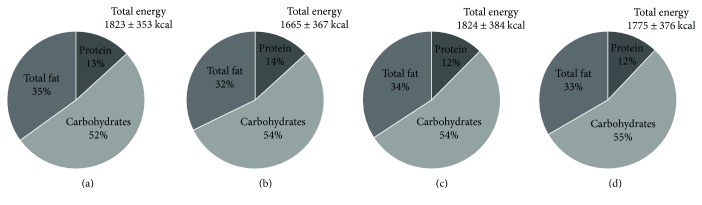
Changes in dietary profile after intervention. Showing dietary profile. (a) Preintervention in the intervention group (*n*, 38). (b) Postintervention in the intervention group (*n*, 25). (c) Preintervention in the control group (*n*, 38). (d) Postintervention in the control group (*n*, 25).

The subsample assessment showed a significant reduction in the sedentary lifestyle. More individuals converted from sedentary to more active lifestyle (67% to 55%) in the intervention group (Figure 4(b)) as compared to the control group (69% to 65%) (Figure 4(d)). Dietary behaviour also improved in terms of decrease in total calorie intake and fat consumption (Figure 5(b)).

## 4. Discussion

In this randomized control trial, high prevalence of obesity, abdominal obesity, hypertension, dyslipidemia, and metabolic syndrome was observed in relatively young and overweight individuals working in corporate setups in North India. Further, multicomponent interventions based on face-to-face interactions as well as with the use of digital platform showed significant decrease in weight, BMI, waist circumference, hip circumference, W-HR, both peripheral and central skinfolds, and triglycerides and increase in HDL-c. It is important to note change in clustering of risk profile status from multiple clustering (3–5 per individual) to lesser clustering.

Worksite intervention programs have been rarely done in India. In particular, a closely related intervention, tobacco control at worksites, has been eminently successful, backed by tight legal regulations in India. A recent study on 20 worksites in manufacturing sector successfully demonstrated doubling of the 6-month smoking cessation rates among workers in the intervention worksites compared to those in the control sites [18].

In a study carried out in 10 different industrial sites representing multiple regions of India, the lifestyle-based intervention surveys and cohort analysis showed a significant relative reduction in the cardiovascular risk factors except in serum triglycerides in the intervention group versus the control group [19]. Further, in South India, a healthy workplace model was evaluated in workers of a software industry. In this study, high levels of risk factors, obesity (55%), and hypertension (15%) were recorded. Based on these data, modifications in the workplace targeting physical and psychosocial work environment were suggested by the authors [20]. In the current study, we show that clustering of multiple risk factors was reduced, along with weight, abdominal, and generalized adiposity, which may contribute to prevention of diabetes and cardiovascular disease. Most importantly, behaviour changes were seen in our study after intervention, with significant improvements in physical activity and change in diet towards healthier options.

We assessed skinfold thicknesses at different sites, which has not been previously done in any such study. These skinfold measurements show magnitude and distribution of subcutaneous adiposity in an individual. It is important to note that subcutaneous adipose tissue is high in Asian Indians [1]. The importance of skinfold measurements, specifically subscapular/triceps ratio (central obesity), was initially shown by Haffner et al. [21] in Mexican Americans. In this study, using multiple logistic regressions with age, ethnicity, BMI, and central obesity as covariates, the overall obesity was positively associated with T2DM prevalence in both sexes but central obesity was related to prevalence of T2DM only in women. In another analysis on Mexican Americans by the same group, subscapular/triceps ratio and W-HR both were associated with high rates of diabetes, low HDL-c levels, and high triglyceride level [22]. It is important to note that in the current trial, the most impressive changes after the intervention were observed in skinfold thicknesses. Specifically, the sum of subscapular and suprailiac skinfold (indicative of truncal subcutaneous adiposity) was significantly reduced in intervention group (7.14 mm) as compared to the control group (1.65 mm). Further, we also show that 28% participants in the intervention group had decreased sum of skinfolds below the threshold that signifies insulin resistance versus 9% in the control group. Importance of excess truncal subcutaneous tissue in the context of dysmetabolic state in Asian Indians has been emphasized previously. Specifically, excess truncal subcutaneous adipose tissue (as shown by central skinfolds) showed close correlation with insulin resistance and metabolic syndrome in Asian Indians [23]. In a previous study on Asian Indian adolescents, using binary recursive analysis, we showed that excess sums of the four skinfolds (central and peripheral) are important determinants of insulin resistance [24]. In a recent comparative study between nondiabetic Asian Indians with nonalcoholic fatty liver disease (NAFLD) versus nondiabetic individuals without NAFLD, subscapular, suprailiac, and central skinfolds were significantly higher in the former [25], signifying relationship of truncal subcutaneous adiposity to NAFLD, a central facet of metabolic syndrome. Interestingly, as compared to White Caucasians, South Asians have larger adipocytes from subcutaneous adipose tissue, associated with significant insulin resistance. Further, subcutaneous adipocyte area was higher in Asian Indians with increased body fat percentage and hepatic fat as compared to White Caucasians with similar BMI [26]. Finally, subcutaneous abdominal adipose tissue mRNA expression was significantly higher for genes associated with inflammation and CD68, MAC1, and MCP1 in Asian Indians compared with whites, meaning increased inflammation generated from subcutaneous adipocytes in the former [27].

It is encouraging to report significant decrease in triglycerides after the intervention (11.0 mg/dl in the intervention group versus 0.12 mg/dl in the control group) which is different to previous worksite intervention study in India [19]. Atherogenic dyslipidemia is particularly common in South Asians and has been shown to have a strong association with T2DM, metabolic syndrome, and coronary heart disease (CHD). Interestingly, triglyceride levels have been shown to rise steeply before 20 years age to maximally in 30–39 years age in Asian Indians [3]. In particular, low HDL levels are common in Asian Indians and are lower than that seen in Whites [1]. Further, HDL particles also appear to be smaller, dysfunctional, and proatherogenic in South Asians [1]. Whether such betterment of serum triglycerides with increase in HDL-c levels leads to any cardiovascular benefit continues to be researched. In a recent study on 28,318 members (aged 30 to 90 years), the presence of atherogenic dyslipidemia was associated with the highest age-adjusted CHD events/1000 patient years after multiple adjustments and even in those with LDL-c < 100 mg/dl [28]. Hence, any such decrease in triglycerides and increase in HDL-c levels after intervention, as has been shown in our study, are likely to decrease cardiovascular risk. Interestingly, increased thickness of truncal adipose tissue, as seen in our study, is typical of Asian Indians and could be strongly related to high triglycerides and low HDL-c levels from early age [29].

Finally, there are a few limitations of our study; the study was of a short-term duration. The future trials should include more number of participants with lesser number of subjects who are lost to follow-ups and should be conducted over a longer period of time.

Furthermore, physical activity and dietary habit data could be collected in larger number of individuals. Also, more measures of glycaemia (glycosylated hemoglobin) and inflammatory markers (high sensitivity C-reactive protein) could be included. The strength of our study is the design (randomized trial) and clinical measurement of truncal subcutaneous adiposity, which is important adiposity measure in Asian Indians.

## 5. Conclusion

In conclusion, this multicomponent lifestyle intervention study conducted in overweight worksite individuals with the cardiometabolic risk factors was successful in achieving of reduction in weight, excess subcutaneous fat, and cardiometabolic risk factors after 6 months of active intervention. Results of this study are reasonably convincing to encourage other worksites in India to implement similar kind of multicomponent interventions.

## Figures and Tables

**Table 1:** Baseline characteristics.

Variable	Intervention Frequency in % or mean ± SD (*n* = 156)	Control Frequency in % or mean ± SD (*n* = 111)
Mean age in years ± SD	35.8 ± 7.6	39.0 ± 8.7
Males	87.9	82.5
Females	12.1	17.5
Marital status		
Married	85.7	89.3
Single education	14.3	10.7
Senior secondary	4.4	2.3
Graduate	47.8	45.2
Postgraduate	46.7	52.0
Doctorate	1.1	0.6
Household income		
More than 75(000) INR^∗^	73.6	65.5
65–75 (000) INR	9.9	6.8
55–65 (000) INR	4.9	7.9
45–55 (000) INR	6.0	9.6
Less than 45 (000) INR	5.4	10.2
Tobacco user		
Never	79.1	76.8
Current	15.4	15.8
Past	5.5	7.3
Alcohol user		
Never	45.1	42.9
Current	47.3	46.3
Past	7.7	10.7

^∗^INR: Indian national rupee.

**Table 2:** Prevalence (%) of high/abnormal values of risk factors^∗^ at baseline.

Variable	Intervention	Control
Obesity	90.9	80.2
	M: 91.9; F: 84.2	M:77.9; F:93.8
Sum of skinfolds (mm)	70.3	75.9
	M: 67.6; F: 89.5	M: 71.7; F: 100.0
Waist circumference (cm)	93.5	84.3
	M: 94.9; F: 84.2	M:82.6; F:93.8
Waist-hip ratio	91.1	90.7
	M: 92.6; F:78.9	M:91.3; F:87.5
Systolic blood pressure (mmHg)	31.2	31.0
	M:34.8; F:5.3	M:31.8; F: 26.7
Diastolic blood pressure (mmHg)	40.3	44.0
	M: 43.7; F: 15.8	M: 48.2; F: 20
Fasting blood glucose (mg/dl)	25.7	30.6
	M: 29.2; F: 0.0	M: 30.6; F:30.8
Total cholesterol (mg/dl)	33.1	29.4
	M:32.3; F: 38.9	M: 32.2; F:13.3
High-density lipoprotein cholesterol (mg/dl)	56.8	63.7
	M: 55.4; F: 66.7	M: 62.1; F:73.3
Low-density lipoprotein cholesterol (mg/dl)	72.3	74.5
	M: 73.8; F: 61.1	M: 73.6; F:80.0
Serum triglycerides (mg/dl)	37.2	33.3
	M:40.0; F:16.7	M: 35.6; F:20.0

All values in percentages. ^∗^See text for definitions of abnormal values.

**Table 3:** Mean values of anthropometric, clinical, and biochemical profiles at baseline.

Parameter	Intervention		Control		Intervention-control (Δ)	Statistical significance of intervention versus control at baseline	
	*n*	Mean ± SD	*n*	Mean ± SD	Mean difference	*z* values	*p* values
Weight (kg)	156	81.67 ± 10.72	111	80.89 ± 12.24	0.77	0.536	0.592
BMI (kg/m^2^)	156	28.21 ± 2.89	111	28.20 ± 3.59	0.02	0.039	0.969
Waist circumference (cm)	155	98.90 ± 8.58	111	98.89 ± 9.81	0.25	0.218	0.827
Hip circumference(cm)	155	103.70 ± 5.72	111	103.84 ± 7.83	−0.14	0.161	0.872
Waist-hip ratio	155	0.95 ± 0.06	111	0.96 ± 0.06	−0.01	0.798	0.425
Skinfolds (mm)							
Biceps	155	8.69 ± 4.02	108	9.26 ± 3.86	−0.56	1.148	0.251
Triceps	155	16.64 ± 5.69	108	16.33 ± 5.66	0.32	0.443	0.658
Subscapular	155	22.83 ± 6.15	108	22.88 ± 6.08	−0.05	0.067	0.947
Suprailiac	155	18.11 ± 6.34	108	18.80 ± 6.76	−0.69	0.831	0.406
Sum of peripheral skinfolds	155	25.3 ± 10.13	108	25.6 ± 9.72	−0.25	0.201	0.841
Sum of central skinfolds	155	40.9 ± 12.27	108	41.7 ± 12.93	−0.80	0.504	0.614
Sum of 4 skinfolds (∑4SF)	155	66.28 ± 18.74	108	67.27 ± 18.84	−0.99	0.419	0.675
Systolic blood pressure (mmHg)	154	122.04 ± 13.04	100	124.67 ± 13.40	−2.63	1.547	0.122
Diastolic blood pressure (mmHg)	154	82.61 ± 8.95	100	83.87 ± 9.86	−1.25	1.025	0.305
Fasting blood glucose (mg/dl)	148	96.52 ± 12.29	102	98.03 ± 10.41	−1.51	−0.998	0.318
Total cholesterol (mg/dl)	148	187.74 ± 36.00	102	181.63 ± 36.72	6.10	1.302	0.193
High-density lipoprotein cholesterol (mg/dl)	148	40.90 ± 9.51	102	40.10 ± 8.32	0.80	0.700	0.484
Low-density lipoprotein cholesterol (mg/dl)	148	118.00 ± 26.98	102	117.57 ± 32.38	0.43	0.111	0.912
Serum triglycerides (mg/dl)	148	145.74 ± 80.4	102	137.31 ± 62.48	8.43	0.931	0.352

**Table 4:** Changes in values of body composition, blood pressure, and metabolic factors after intervention.

Variable		*N*	Intervention mean ± SD	*p* value	*N*	Control mean ± SD	*p* value	Change in intervention versus control Δ1-Δ2	*p* value
Weight (kg)	Baseline Follow-up	156 156	81.67 ± 10.72 80.07 ± 10.50	*p* < 0.001	111	80.89 ± 12.24 80.51 ± 12.31	*p* = 0.0455	1.22	*p* < 0.001
	Difference		1.60 ± 2.76			0.38 ± 2.03			
BMI (kg/m^2^)	Baseline Follow-up	156 156	28.21 ± 2.89 27.6 ± 2.8	*p* < 0.001	112	28.20 ± 3.59 28.04 ± 3.62	*p* = 0.0278	0.39	*p* < 0.001
	Difference		0.55 ± 0.96		112	0.16 ± 0.75			
Waist circumference (cm)	Baseline Follow-up	155 155	98.90 ± 8.58 97.04 ± 8.18	*p* < 0.001	111	98.89 ± 9.81 99.20 ± 9.81	*p* = 0.1902	2.11	*p* < 0.001
	Difference		1.87 ± 3.49		111	−0.31 ± 9.81			
Hip circumference (cm)	Baseline Follow-up	155 155	103.70 ± 5.72 102.46 ± 5.69	*p* < 0.001	111	103.84 ± 7.83 103.60 ± 8.08	*p* = 0.3222	1.00	*p* < 0.001
	Difference		1.23 ± 2.94		111	0.23 ± 2.49			
Waist-hip ratio	Baseline Follow-up	155 155	0.954 ± 0.062 0.947 ± 0.061	*p* = 0.0239	111	0.950 ± 0.064 0.955 ± 0.062	*p* = 0.064	0.01	*p* < 0.001
	Difference		0.007 ± 0.029		111	0.005 ± 0.028			
Skinfold biceps (mm)	Baseline Follow-up	155 155	8.69 ± 4.02 6.85 ± 2.95	*p* < 0.001	108	9.26 ± 3.86 8.31 ± 3.68	*p* < 0.001	0.89	*p* < 0.001
	Difference		1.84 ± 2.26		108	0.95 ± 1.57			
Triceps (mm)	Baseline Follow-up	155 155	16.64 ± 5.69 13.11 ± 5.19	*p* < 0.001	108	16.33 ± 5.66 15.43 ± 5.41	*p* < 0.001	2.64	*p* < 0.001
	Difference		3.54 ± 3.67		108	0.90 ± 2.70			
Subscapular (mm)	Baseline Follow-up	155 155	22.83 ± 6.15 19.45 ± 4.91	*p* < 0.0001	108	22.88 ± 6.08 21.87 ± 5.22	*p* < 0.001	2.36	*p* < 0.001
	Difference		3.38 ± 4.21		108	1.01 ± 3.35			
Suprailiac (mm)	Baseline Follow-up	155 155	18.11 ± 6.34 14.35 ± 5.18	*p* < 0.001	108	18.80 ± 6.76 18.16 ± 6.31	*p* = 0.0601	3.12	*p* < 0.001
	Difference		3.76 ± 3.79		108	0.64 ± 3.53			
Central skinfolds (mm)^∗^	Baseline Follow-up	155 155	40.94 ± 12.27 33.8 ± 10.14	*p* < 0.001	108	41.68 ± 12.93 40.03 ± 11.60	*p* = 0.0102	5.40	*p* < 0.001
	Difference		7.14 ± 8.00		108	1.65 ± 6.87			
Peripheral skinfolds (mm)^∗∗^	Baseline Follow-up	155 155	25.29 ± 10.13 19.87 ± 8.19	*p* < 0.001	108	25.59 ± 9.52 23.74 ± 9.10	*p* < 0.001	3.53	*p* < 0.001
	Difference		5.42 ± 5.92		108	1.84 ± 5.92			
∑4SF skinfolds (mm)^∗∗∗^	Baseline Follow-up	155 155	66.28 ± 18.74 53.77 ± 15.37	*p* < 0.001	108	67.27 ± 18.84 63.77 ± 17.39	*p* < 0.001	9.02	*p* < 0.001
	Difference		12.51 ± 10.38		108	3.50 ± 8.18			
SBP (mmHg)	Baseline Follow-up	154 154	122.04 ± 13.04 121.39 ± 12.64	*p* = 0.4413	100	124.67 ± 13.40 124.89 ± 12.87	*p* = 0.8572	0.86	*p* = 0.5505
	Difference		0.65 ± 10.44		100	−0.22 ± 11.77			
DBP (mmHg)	Baseline Follow-up	154 154	82.61 ± 8.95 80.29 ± 9.92	*p* < 0.001	100	83.87 ± 9.86 80.89 ± 7.83	*p* = <0.001	−0.65	*p* = 0.5734
	Difference		2.32 ± 7.57		100	2.98 ± 9.88			
FBG (mg/dl)	Baseline Follow-up	148 148	96.52 ± 12.29 93.63 ± 11.64	*p* < 0.001	102	98.03 ± 10.41 95.77 ± 9.49	*p* < 0.001	0.63	*p* = 0.4319
	Difference		2.89 ± 6.48		102	2.26 ± 5.88			
TC (mg/dl)	Baseline Follow-up	148 148	187.74 ± 36.00 182.96 ± 36.35	*p* = 0.0164	102	181.63 ± 36.72 184.40 ± 32.11	*p* = 0.2301	7.55	*p* = 0.0134
	Difference	148	4.78 ± 24.17		102	−2.77 ± 23.37			
HDL-c (mg/dl)	Baseline Follow-up	148 148	40.90 ± 9.51 43.10 ± 8.86	*p* < 0.001	102	40.10 ± 8.32 40.49 ± 8.10	*p* = 0.4179	−1.81	*p* = 0.0051
	Difference		−2.20 ± 5.37		102	−0.39 ± 4.79			
LDL-c (mg/dl)	Baseline Follow-up	148 148	118.00 ± 26.98 116.60 ± 30.54	*p* = 0.4654	102	117.57 ± 32.38 118.98 ± 29.95	*p* = 0.5419	2.81	*p* = 0.3498
	Difference		1.40 ± 23.20		102	−1.41 ± 23.42			
TG (mg/dl)	Baseline Follow-up	148 148	145.74 ± 80.41 134.67 ± 71.09	*p* = 0.0039	102	137.31 ± 62.48 137.19 ± 64.46	*p* = 0.9681	10.95	*p* = 0.0218
	Difference		11.07 ± 46.56		102	0.12 ± 28.85			

^∗^Central skinfold is the sum of subscapular and suprailiac skinfolds. ^∗∗^Peripheral skinfold is the sum of biceps and triceps skinfolds. ^∗∗∗^∑4SF is the sum of all 4 skinfolds (biceps, triceps, subscapular, and suprailiac); SBP: systolic blood pressure; DBP: diastolic blood pressure; FBG: fasting blood glucose; TC: total cholesterol; HDL-c: high-density lipoprotein cholesterol; LDL-c: low-density lipoprotein cholesterol; TG: serum triglycerides.

**Table 5:** Changes in abnormal profile of various factors to normal after intervention.

Parameters	Intervention		Control		Statistical significance of change in intervention versus change in control	
Risk factors	Pre	Post	Pre	Post	*z* values	*p* values
Obesity (BMI ≥ 25 kg/m^2^)	90.9	85.6	80.2	86.5	1.830	0.0673
Sum of four skinfolds (∑4SF ≥ 53.8 mm)	70.3	42.6	75.9	66.7	2.263	0.0237
Waist circumference (WC ≥ 90 cm M, ≥ 80 cm F)	93.5	92.3	84.3	85.2	0.089	0.0896
Waist-hip ratio (≥0.9 M, ≥ 0.8 F)	91.0	91.0	90.7	90.7	0.175	0.8611
SBP ≥ 130 mmHg	31.2	24.7	31	32	0.901	0.3675
DBP ≥ 85 mmHg	40.3	30.5	44.0	30.0	0.492	0.6229
FBG ≥ 100 mg/dl	25.7	18.9	30.6	22.8	0.005	0.9957
Total cholesterol ≥ 200 mg/dl	33.1	27.7	29.4	32.4	0.995	0.3195
HDL (≤40 mg/dl in M and ≤50 mg/dl in F)	56.8	40.5	63.7	59.8	1.382	0.1669
LDL (≥100 mg/dl)	72.3	69.6	74.5	74.5	0.335	0.7376
TG (≥150 mg/dl)	37.2	27.0	33.3	38.2	1.748	0.0805

All values are in percentages; SBP: systolic blood pressure; DBP: diastolic blood pressure; FBG: fasting blood glucose; HDL-c: high-density lipoprotein cholesterol; LDL-c: low-density lipoprotein cholesterol; TG: serum triglycerides; M: males; F: females.
